# Supplementary figures and images for: Developing a machine learning model to map new-build gentrification: A mixed-methods approach
Source: PLoS One. 2026 Jan 30;21(1):e0341844. doi: 10.1371/journal.pone.0341844 (PMC12858069; doi:10.1371/journal.pone.0341844)

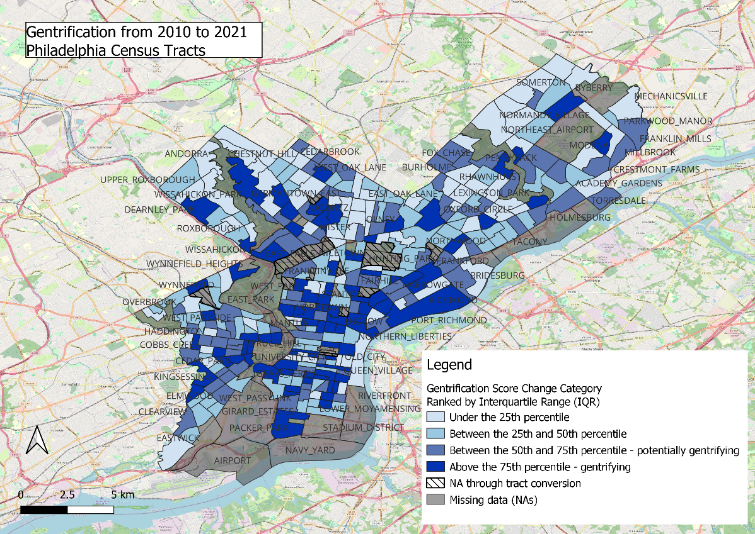

Supplement: S1 Fig — Principal Components Analysis (PCA) output indicating gentrification-indicative socioeconomic change. Census tract boundaries generated using data from Tim Wisniewski (2016), licensed under the MIT License. © Tim Wisniewski. Source: https://opendataphilly.org/datasets/census-tracts/. Basemap data © OpenStreetMap contributors, licensed under the Open Data Commons Open Database License (ODbL). Source: https://www.openstreetmap.org. (TIF) [file pone.0341844.s001.tif]

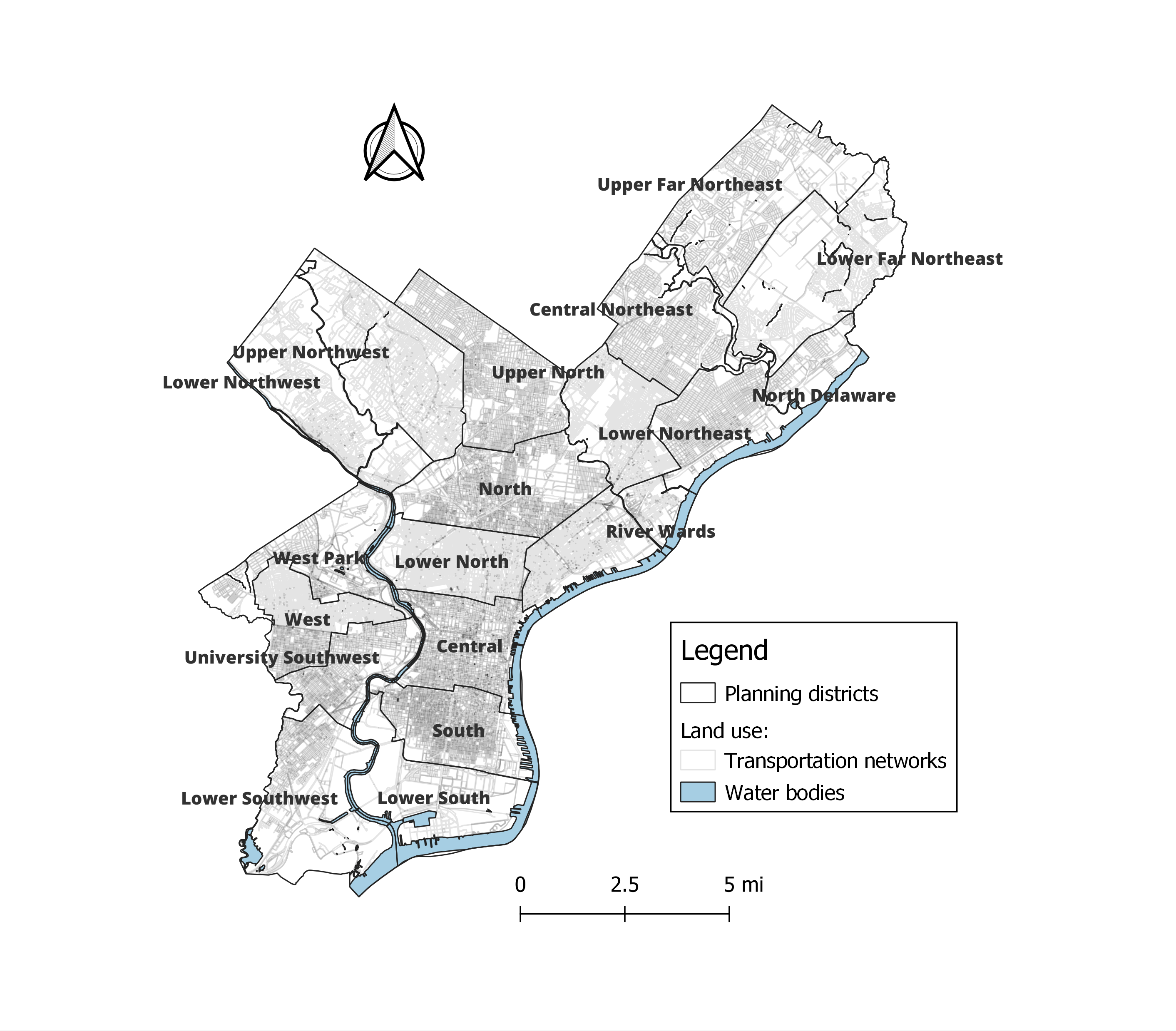

Supplement: S2 Fig — Reference map for Philadelphia’s neighborhood boundaries. Adapted from Philadelphia Neighborhoods by Robert Cheetham (2014). © Robert Cheetham, licensed under CC BY 4.0. Source: https://opendataphilly.org/datasets/philadelphia-neighborhoods/. (TIFF) [file pone.0341844.s002.tiff]
