# Supplementary material for: Developing a machine learning model to map new-build gentrification: A mixed-methods approach
Source: PLoS One. 2026 Jan 30;21(1):e0341844. doi: 10.1371/journal.pone.0341844 (PMC12858069; doi:10.1371/journal.pone.0341844)
Supplement: S1 Table — PCA output as importance of PC components for the year 2010 ACS Census variables. (DOCX) [file pone.0341844.s003.docx]

**Table S1.** **Principal Components Analysis (PCA) Summary Statistics for 2010.** PCA output as importance of PC components for the year 2010 ACS Census variables

|  | PC1 | PC2 | PC3 | PC4 |
| --- | --- | --- | --- | --- |
| Standard deviation | 1.715 | 0.7907 | 0.5748 | 0.32282 |
| Proportion of variance | 0.735 | 0.1563 | 0.0826 | 0.02605 |
| Cumulative proportion | 0.735 | 0.8913 | 0.9739 | 1.00000 |
