# Supplementary material for: Developing a machine learning model to map new-build gentrification: A mixed-methods approach
Source: PLoS One. 2026 Jan 30;21(1):e0341844. doi: 10.1371/journal.pone.0341844 (PMC12858069; doi:10.1371/journal.pone.0341844)
Supplement: S2 Table — PCA output as importance of PC components for the year 2021 ACS Census variables. (DOCX) [file pone.0341844.s004.docx]

**Table S2.** **Principal Components Analysis (PCA) Summary Statistics for 2021.** PCA output as importance of PC components for the year 2021 ACS Census variables

|  | PC1 | PC2 | PC3 | PC4 |
| --- | --- | --- | --- | --- |
| Standard deviation | 1.8237 | 0.61299 | 0.46613 | 0.28482 |
| Proportion of variance | 0.8315 | 0.09394 | 0.05432 | 0.02028 |
| Cumulative proportion | 0.8315 | 0.92540 | 0.97972 | 1.00000 |
