# Supplementary material for: Developing a machine learning model to map new-build gentrification: A mixed-methods approach
Source: PLoS One. 2026 Jan 30;21(1):e0341844. doi: 10.1371/journal.pone.0341844 (PMC12858069; doi:10.1371/journal.pone.0341844)
Supplement: Attachment — Submitted filename: Response to the editor.docx [file pone.0341844.s006.docx]

Thank you to the editor for taking the time to evaluate our manuscript. We address the comments below:

***1. Please ensure that your manuscript meets PLOS ONE's style requirements, including those for file naming. The PLOS ONE style templates can be found at***

[***https://journals.plos.org/plosone/s/file?id=wjVg/PLOSOne_formatting_sample_main_body.pdf***](https://journals.plos.org/plosone/s/file?id=wjVg/PLOSOne_formatting_sample_main_body.pdf) ***and***

[***https://journals.plos.org/plosone/s/file?id=ba62/PLOSOne_formatting_sample_title_authors_affiliations.pdf***](https://track.editorialmanager.com/CL0/https:%2F%2Fjournals.plos.org%2Fplosone%2Fs%2Ffile%3Fid=ba62%2FPLOSOne_formatting_sample_title_authors_affiliations.pdf/1/010f01990d7f526b-e9932142-d6b8-4ace-83c6-f12bdbe9e941-000000/HUl3Fq3gBqKz84GVoRk4pzw6BhFkbhSTE5vXyHJDxyE=227)***a***

We ensured that the PLOS ONE style requirements were met, including those for file naming. We were able to find specific guidelines to naming the supplementary materials files and have revised those names accordingly. We could not find specific information about naming the other main submission files, so please let us know if there are any errors on that end.

***2. In the ethics statement in the Methods, you have specified that verbal consent was obtained. Please provide additional details regarding how this consent was documented and witnessed, and state whether this was approved by the IRB.***

We specify how consent was documented and witnessed on lines 287-289 in the section “Development of an auditing protocol with focus group insights”. Details about IRB approval are provided on lines 276-278. These line numbers reference the manuscript version with track changes on.

***3. Please note that PLOS One has specific guidelines on code sharing for submissions in which author-generated code underpins the findings in the manuscript. In these cases, we expect all author-generated code to be made available without restrictions upon publication of the work. Please review our guidelines at*** [***https://journals.plos.org/plosone/s/materials-and-software-sharing#loc-sharing-code***](https://journals.plos.org/plosone/s/materials-and-software-sharing#loc-sharing-code) ***and ensure that your code is shared in a way that follows best practice and facilitates reproducibility and reuse.***

Thank you for your comment. We have shared the minimal dataset via GitHub. The link to the data repository is as follows: <https://github.com/niiquaye70/Mapping-New-Build-Gentrification-with-Machine-Learning/tree/main/data>. The DOI is <https://doi.org/10.5281/zenodo.17352508>.

***4. We note that your Data Availability Statement is currently as follows: All relevant data are within the manuscript and in Supporting Information files.***

***Please confirm at this time whether or not your submission contains all raw data required to replicate the results of your study. Authors must share the “minimal data set” for their submission. PLOS defines the minimal data set to consist of the data required to replicate all study findings reported in the article, as well as related metadata and methods (https://journals.plos.org/plosone/s/data-availability#loc-minimal-data-set-definition).***

***For example, authors should submit the following data:***

***- The values behind the means, standard deviations and other measures reported;***

***- The values used to build graphs;***

***- The points extracted from images for analysis.***

***Authors do not need to submit their entire data set if only a portion of the data was used in the reported study.***

***If your submission does not contain these data, please either upload them as Supporting Information files or deposit them to a stable, public repository and provide us with the relevant URLs, DOIs, or accession numbers. For a list of recommended repositories, please see*** [***https://journals.plos.org/plosone/s/recommended-repositories***](https://journals.plos.org/plosone/s/recommended-repositories)***.***

***If there are ethical or legal restrictions on sharing a de-identified data set, please explain them in detail (e.g., data contain potentially sensitive information, data are owned by a third-party organization, etc.) and who has imposed them (e.g., an ethics committee). Please also provide contact information for a data access committee, ethics committee, or other institutional body to which data requests may be sent. If data are owned by a third party, please indicate how others may request data access.***

Thank you for your comment. We have shared the minimal dataset via GitHub. The link to the data repository is as follows: <https://github.com/niiquaye70/Mapping-New-Build-Gentrification-with-Machine-Learning/tree/main/data>. The DOI is <https://doi.org/10.5281/zenodo.17352508>. We have acquired an open-source MIT license as follows:

MIT License

Copyright (c) 2023 NSF #2312047 and #2312048 Projects

Permission is hereby granted, free of charge, to any person obtaining a copy of this software and associated documentation files (the "Software"), to deal in the Software without restriction, including without limitation the rights to use, copy, modify, merge, publish, distribute, sublicense, and/or sell copies of the Software, and to permit persons to whom the Software is furnished to do so, subject to the following conditions:

The above copyright notice and this permission notice shall be included in all copies or substantial portions of the Software.

THE SOFTWARE IS PROVIDED "AS IS", WITHOUT WARRANTY OF ANY KIND, EXPRESS OR IMPLIED, INCLUDING BUT NOT LIMITED TO THE WARRANTIES OF MERCHANTABILITY, FITNESS FOR A PARTICULAR PURPOSE AND NONINFRINGEMENT. IN NO EVENT SHALL THE AUTHORS OR COPYRIGHT HOLDERS BE LIABLE FOR ANY CLAIM, DAMAGES OR OTHER LIABILITY, WHETHER IN AN ACTION OF CONTRACT, TORT OR OTHERWISE, ARISING FROM, OUT OF OR IN CONNECTION WITH THE SOFTWARE OR THE USE OR OTHER DEALINGS IN THE SOFTWARE.

We decline to share the focus group data because it contains sensitive locational information about participants' neighborhoods. As part of our consent process, we ensured participants that the information they shared with us would remain confidential and that all information shared beyond the research team would be de-identified. Because the information they shared with us includes specific references to neighborhood characteristics, we are unable to fully de-identify the focus group notes. Moreover, we believe the research can be replicated without this data.

***5. Please include your full ethics statement in the ‘Methods’ section of your manuscript file. In your statement, please include the full name of the IRB or ethics committee who approved or waived your study, as well as whether or not you obtained informed written or verbal consent. If consent was waived for your study, please include this information in your statement as well.***

We included the full ethics statement in the “Development of an auditing protocol with focus group insights” subsection of the “Methods” section. The full name of the IRB committee is provided on lines 258-259, and the information about consent is provided on lines 276-278.

***6. We note that Figure 4 and S2 in your submission contain map/satellite images which may be copyrighted. All PLOS content is published under the Creative Commons Attribution License (CC BY 4.0), which means that the manuscript, images, and Supporting Information files will be freely available online, and any third party is permitted to access, download, copy, distribute, and use these materials in any way, even commercially, with proper attribution. For these reasons, we cannot publish previously copyrighted maps or satellite images created using proprietary data, such as Google software (Google Maps, Street View, and Earth). For more information, see our copyright guidelines:*** [***http://journals.plos.org/plosone/s/licenses-and-copyright***](http://journals.plos.org/plosone/s/licenses-and-copyright)***.***

***We require you to either (1) present written permission from the copyright holder to publish these figures specifically under the CC BY 4.0 license, or (2) remove the figures from your submission.***

Figure 4 was created by the authors using the ArcGIS geospatial mapping tool and data acquired from a publicly available municipal dataset “Licenses and Inspections Building and Zoning Permits” via *OpenDataPhilly.* Information on accessing this data is provided on lines 346-251. The map also features data from our machine learning model. Lastly, Figure 4 also features a minimal boundary shapefile of census tract divisions that was downloaded via *OpenDataPhilly*. To the best of our knowledge, this figure does not require a copyright statement, but please let us know if any of these data sources pose any copyright issues that we overlooked.

Figure S2 (now S1) employs an overlay OpenStreetMap base map from the QGIS geospatial mapping tool application, a census tract boundary shapefile from *OpenDataPhilly,* and data from our Principal Components Analysis (PCA) that draws on American Community Survey (ACS) data. To the best of our knowledge and after examining the data copyright stipulations of these sources, none of these datasets and imagery are restricted access by copyright rules. We add more detail about the sources of our data in the figure captions for both figures. The new Figure S2 also does not infringe on any copyright laws, with all shapefiles in the map image deriving from open-source *OpenDataPhilly* datasets for Land Use and Neighborhood Planning District boundaries.

***7. We note that Figures 1-3, 6 and S1 in your submission contain copyrighted images. All PLOS content is published under the Creative Commons Attribution License (CC BY 4.0), which means that the manuscript, images, and Supporting Information files will be freely available online, and any third party is permitted to access, download, copy, distribute, and use these materials in any way, even commercially, with proper attribution. For more information, see our copyright guidelines:*** [***http://journals.plos.org/plosone/s/licenses-and-copyright***](http://journals.plos.org/plosone/s/licenses-and-copyright)***.***

***We require you to either (1) present written permission from the copyright holder to publish these figures specifically under the CC BY 4.0 license, or (2) remove the figures from your submission.***

Figure 1, 2, 3, 6, and S1 employ Google Street View (GSV) imagery. In order to comply with copyright rules, we add personally taken photographs of locations in Fishtown that are comparable to the original street view pictures for illustrative purposes. We fully remove Figure 6 and Figure S1. We additionally add in detail to each figure caption, stipulating that all images comply with copyright rules.

***8. We notice that your supplementary figures are uploaded with the file type 'Figure'. Please amend the file type to 'Supporting Information'. Please ensure that each Supporting Information file has a legend listed in the manuscript after the references list.***

We amended the file type to Supporting Information for the supplementary figures. We ensured that the Supporting Information file had a legend listed in the manuscript after the references list.

***9. If the reviewer comments include a recommendation to cite specific previously published works, please review and evaluate these publications to determine whether they are relevant and should be cited. There is no requirement to cite these works unless the editor has indicated otherwise.***

There were no recommendations to cite previously published work.

***10. Please review your reference list to ensure that it is complete and correct. If you have cited papers that have been retracted, please include the rationale for doing so in the manuscript text, or remove these references and replace them with relevant current references. Any changes to the reference list should be mentioned in the rebuttal letter that accompanies your revised manuscript. If you need to cite a retracted article, indicate the article’s retracted status in the References list and also include a citation and full reference for the retraction notice.***

We have reviewed the reference list to ensure that it is complete and correct.
